# The anatomy of the palate in Early Triassic *Chaohusaurus brevifemoralis* (Reptilia: Ichthyosauriformes) based on digital reconstruction

**DOI:** 10.7717/peerj.11727

**Published:** 2021-07-06

**Authors:** Ya-Lei Yin, Cheng Ji, Min Zhou

**Affiliations:** 1 Department of Geology and Geological Museum, Peking University, Beijing, China; 2 State Key Laboratory of Palaeobiology and Stratigraphy, Nanjing Institute of Geology and Palaeontology and Center for Excellence in Life and Paleoenvironment, Chinese Academy of Sciences, Nanjing, China

**Keywords:** *Chaohusaurus brevifemoralis*, Early Triassic, Palate, Ichthyosauriformes, CT scanning

## Abstract

The palatal anatomy of ichthyosauriforms remains largely unknown. Here, the complete palate of the early-branching ichthyosauriform *Chaohusaurus brevifemoralis* is reconstructed and described for the first time with the assistance of high-resolution X-ray computed tomography (CT) scanning on the basis of the three-dimensionally preserved skull of its paratype (GMPKU-P-3086) from the Lower Triassic of South China. The reconstruction reveals new palatal features of *C. brevifemoralis*. The palatine contacts the jugal directly, which is observed in ichthyosauriforms for the first time. A single row of denticles is present on each side of the palate. The vomer exceeds the anterior and posterior margins of the internal naris. The pterygoid is posterior to the internal naris. The epipterygoid is present and the ectopterygoid is absent.

## Introduction

Ichthyosauriformes are a group of the most successful Mesozoic marine reptiles. They originated during the Early Triassic (Motani et al., 2015a) and went extinct during the early Late Cretaceous (Fischer et al., 2016). Compared to the derived members that have been described since 200 years ago, Early Triassic ichthyosauriforms were not well-known due to the incompleteness of the fossil record before the end of the 20th century, and many questions remained unresolved regarding the earliest evolution of this group. During the past several decades, the discovery of abundant Early Triassic ichthyosauriform specimens has greatly improved our understanding on the origin and early adaptation of this group, such as the possibly amphibious *Cartorhynchus* and the potential terrestrial origin of the viviparity supported by *Chaohusaurus* (Motani et al., 2014; Motani et al., 2015a). These new materials revealed a high diversity of ichthyosauriforms during their earliest evolution and resulted in more resolved phylogenetic topology (Motani et al., 2017; Huang et al., 2019).

Compared to the external skull anatomy, palatal morphology and evolution in Early Triassic ichthyosauriforms has received less attention (Callaway, 1989; Maisch & Matzke, 1997a), and the anatomy of the palate in Early Triassic ichthyosauriforms remains poorly known because they were mostly not exposed or preserved (Moon, 2017). No palatal morphology has been known in Nasorostra (Motani et al., 2015a; Jiang et al., 2016), *Parvinatator* (Nicholls & Brinkman, 1995) and *Utatsusaurus* (Cuthbertson, Russell & Anderson, 2014). Incomplete pterygoids and/or palatines are preserved/exposed in *Gulosaurus* (Cuthbertson, Russell & Anderson, 2013a), *Utatsusaurus* (Motani, Minoura & Ando, 1998; Cuthbertson, Russell & Anderson, 2013b) and *Grippia* (Motani, 2000). Specifically, the fragmentary palate of *Utatsusaurus* caused controversial interpretation of the pterygoidal teeth (Motani, 1997; Motani, 1999; Cuthbertson, Russell & Anderson, 2013b; Matsumoto & Evans, 2017). However, some palatal features may be phylogenetically important in basal ichthyosauriforms. For example, the suborbital fenestra is merged with the subtemporal fenestra in Ichthyosauria (Massare & Callaway, 1990) and pterygoidal teeth are present in *Utatsusaurus* but absent in other ichthyosauriforms (Motani, 1999). Moreover, the absence of the ectopterygoid has long been regarded as a synapomorphy of Ichthyopterygia (Motani, 1999; Ji et al., 2015).

*Chaohusaurus*, one of the oldest Mesozoic marine reptiles (Motani, You & McGowan, 1996; Fu et al., 2016), is by far the best known Early Triassic ichthyosauriform (Zhou et al., 2017; Motani et al., 2018; Huang et al., 2019). It retains some terrestrial features, such as head-first viviparity (Motani et al., 2014) and centralia (Motani et al., 2015b). Therefore, this genus provides important information for understanding the aquatic adaptation of ichthyosauriforms, which may shed new light on the evolutionary history of ichthyosauriforms within diapsids. However, the palate of *Chaohusaurus* has only been partially described, although a large number of specimens of this genus have been collected from South China in recent decades. The palate has not been described in *C. zhangjiawanensis* and *C. geishanensis* because it is not observable from the external morphology (Young & Dong, 1972; Chen et al., 2013). The partially exposed pterygoid and/or palatine revealed limited palatal morphology in *C. chaoxianensis* and *C. brevifemoralis* (Maisch, 2001; Zhou et al., 2017; Huang et al., 2019).

Along with the application of many new techniques such as CT scanning in fossil research, the internal morphology can be observed precisely without damaging the material. To date, these techniques have been applied in ichthyosauriform *Platypterygius* (Kear, 2005), *Hauffiopteryx* (Marek et al., 2015), *Protoichthyosaurus* (Lomax, Porro & Larkin, 2019) and *Cartorhynchus* (Huang et al., 2020), which greatly improved our understanding of the skull or other anatomy of these taxa, as well as possibly more historical studies (McGowan, 1989a, 1989b). Under the aid of CT scanning, a detailed description of the complete palate of *C. brevifemoralis* is possible for the first time based on the three-dimensionally preserved skull of its paratype (GMPKU-P-3086). The information derived from this palate improves our understanding on the early evolution of ichthyosauriforms regarding the suborbital fenestra and palatal denticles.

## Material & methods

**Figure 1:**
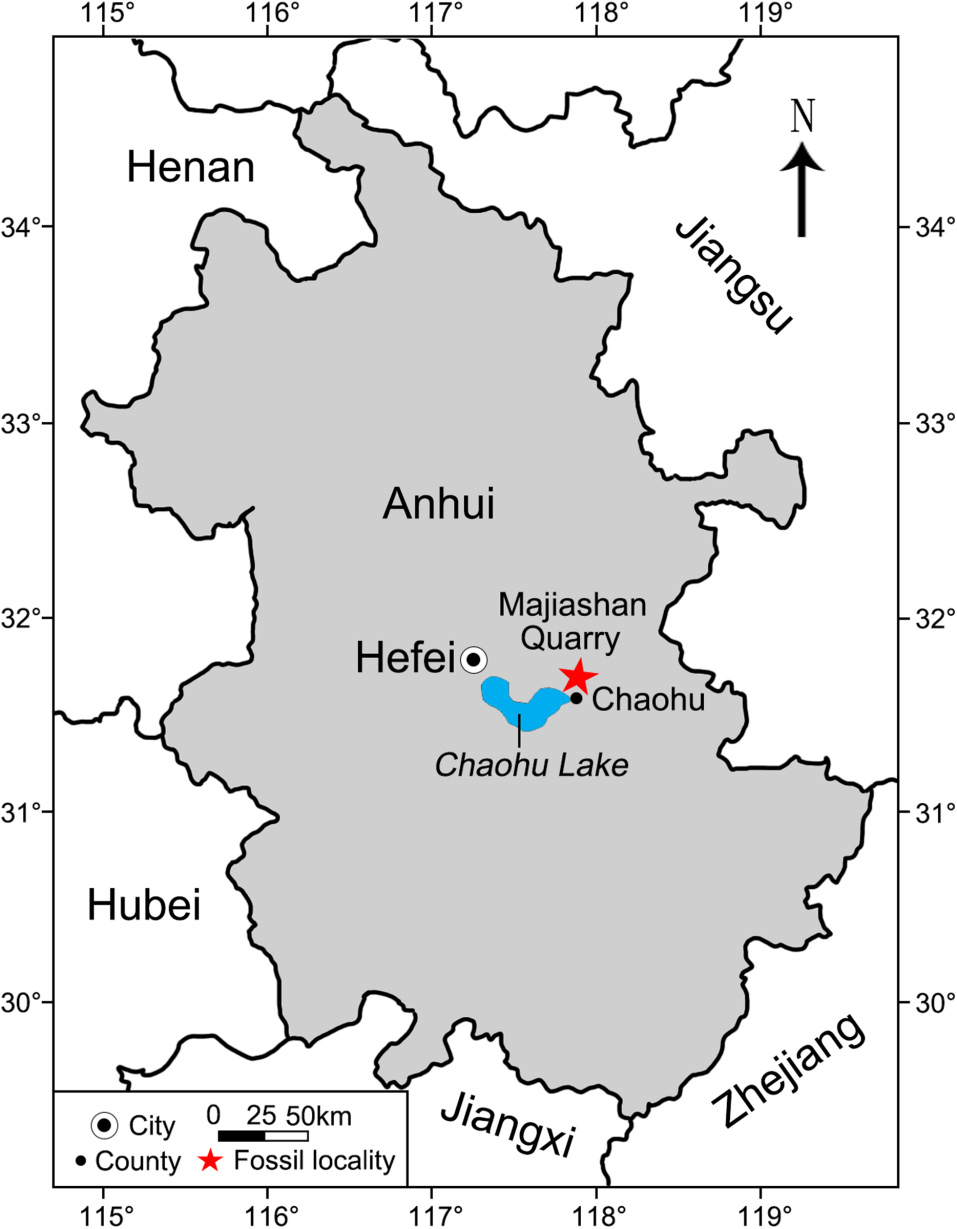
Area map showing the location of Majiashan Quarry (marked by a red asterisk) of *Chaohusaurus brevifemoralis* (GMPKU-P-3086) in Chaohu City, Anhui Province, China.

**Figure 2:**
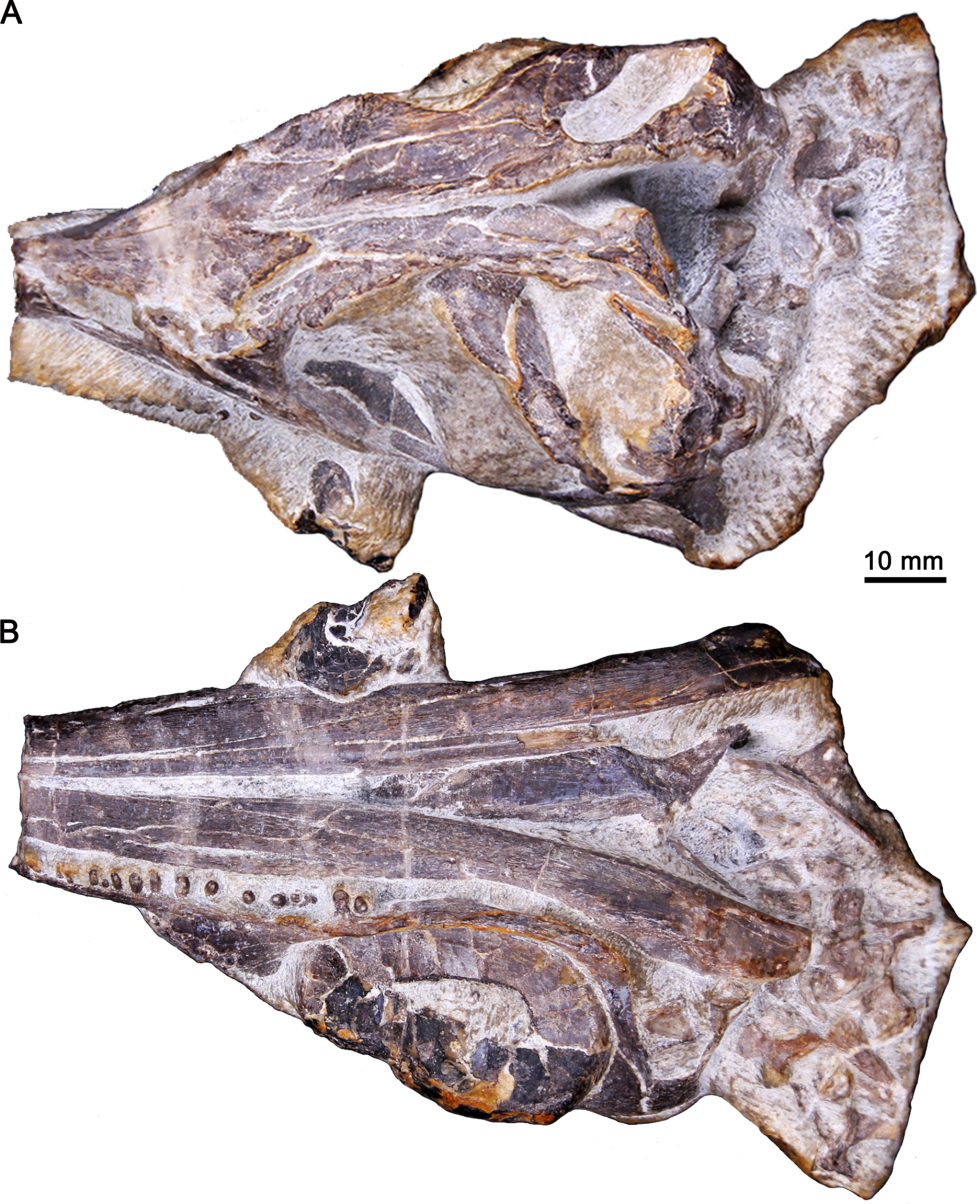
Three-dimensional skull of GMPKU-P-3086. (A) In laterodorsal view. (B) In lateroventral view.

The specimen, GMPKU-P-3086, was discovered in the Nanlinghu Formation (Olenekian, Lower Triassic) at Majiashan Quarry of Chaohu City, Anhui Province, China (Fig. 1), and housed in the Geological Museum of Peking University. The skull of GMPKU-P-3086 was completely separated from the surrounding matrix (Fig. 2). It is comprised of most of the cranium and partial mandibles with four articulated cervical vertebrae. The external skull morphology of GMPKU-P-3086 has been described by Zhou et al. (2017, figs. 3, 4, 5) and Huang et al. (2019, fig. 5). However, the internal skull structure (including the palate) remains nearly unknown.

The skull was scanned using the Nikon XT H 320 LC high-resolution scanner at China University of Geosciences, Beijing. To obtain high-resolution images of the anatomical structures, it was scanned in two sections (anterior and posterior halves), which were then combined, under the same settings along the longitudinal axis. The parameters included 165 kV, 52 μA, no filter, a slice thickness of 34.9 μm, 708-ms exposure time per projection and 3142 projections with one frame per projection. The combination of the data was done by Fiji. Segmentation, measurements, three-dimensional visualization, as well as viewing on image slices were completed using VG Studio Max 2.2 (Volume Graphics, Heidelberg, Germany). Three supplemental videos, including the movie of the 3D reconstructive palate (Video S1; https://www.morphosource.org/concern/media/000355461?locale=en), the movie of palatal CT data (Video S2; https://www.morphosource.org/concern/media/000355466?locale=en) and the horizontal sectional video of the right palatal denticles (Video S3; https://www.morphosource.org/concern/media/000355471?locale=en), are deposited at MorphoSource.

**SYSTEMATIC PALEONTOLOGY**

DIAPSIDA Osborn, 1903

ICHTHYOSAUROMORPHA Motani et al., 2015a

ICHTHYOSAURIFORMES Motani et al., 2015a

*CHAOHUSAURUS Young & Dong, 1972*

*CHAOHUSAURUS BREVIFEMORALIS Huang et al., 2019*

(Figs. 2–5)

**Figure 3:**
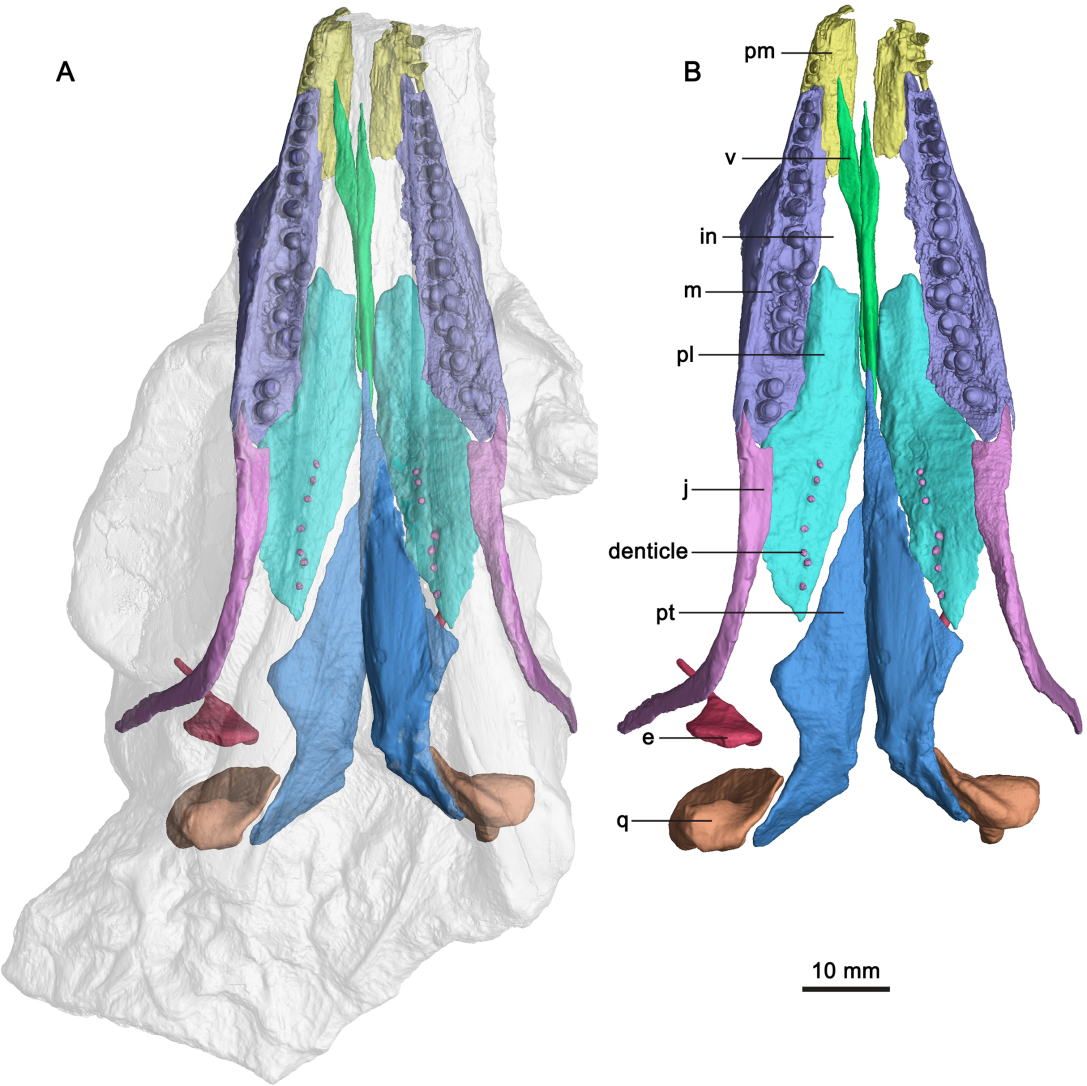
CT-rendered skull elements of GMPKU-P-3086. (A) Semi-transparent skull with the highlight of the palate, premaxilla, maxilla and jugal in ventral view. (B) Palate, premaxilla, maxilla and jugal in ventral view. Abbreviations: e, epipterygoid; in, internal naris; j, jugal; m, maxilla; pl, palatine; pm, premaxilla; pt, pterygoid; q, quadrate; v, vomer.

**Figure 4:**
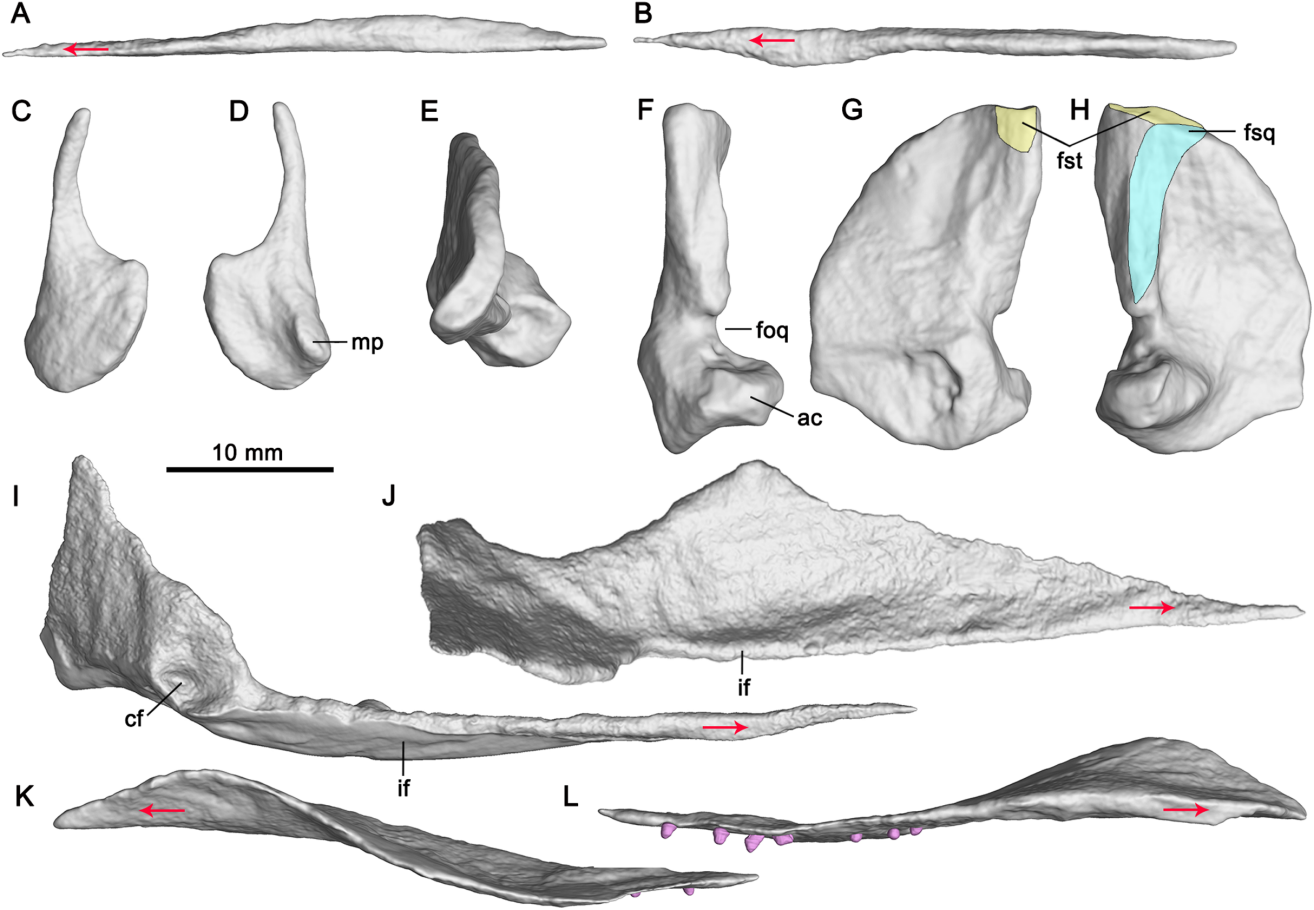
CT-rendered palatal elements of GMPKU-P-3086. Left vomer in lateral (A) and dorsal (B) views; left epipterygoid in lateral (C) and medial (D) views; right quadrate in dorsal (E), posterior (F), medial (G) and lateral (H) views; left pterygoid in medial (I) and dorsal (J) views; left palatine in lateral (K) and medial (L) views. Abbreviations: ac, articular condyle; cf, concave fossa; foq, quadrate foramen; fsq, squamosal facet; fst, supratemporal facet; if, inturned flange; mp, medial process. Red arrows indicate the anterior direction.

**Locality, Horizon and Age**—Majiashan Quarry (Fig. 1), Chaohu City, Anhui Province, China; Nanlinghu Formation, Lower Triassic, ca. 248.53–248.34 Ma (Fu et al., 2016).

**Remarks**—The specimen, GMPKU-P-3086, was originally assigned to *Chaohusaurus chaoxianensis* because it shows that the anterior flange of the humerus is not extensive or uniformly convex, and distal tarsals 1, 2 and 3 are not ossified (Zhou et al., 2017). However, Huang et al. (2019) suggested that these characters were not the autapomorphies of *C. chaoxianensis* and erected a new species of *Chaohusaurus*, *C. brevifemoralis*. GMPKU-P-3086 was attributed to *C. brevifemoralis* as a paratype because it shows the typical diagnostic characters of this species, such as the bifurcation of the caudal peak neural spine, three tarsal ossifications, tibia proximally narrow for trunk length in comparison to *C. chaoxianensis*, and femur short for trunk length in comparison to *C. chaoxianensis* (Huang et al., 2019).

**DESCRIPTION**

The following description focuses exclusively on the palatal region of the skull of the specimen (GMPKU-P-3086). The palate of the specimen is completely preserved (Fig. 3). The internal naris is long and narrow, located just posterior to the level of the external naris. It is bounded by the premaxilla anteriorly, maxilla laterally, palatine posteriorly and vomer medially (Fig. 3). This is different from that of *Mixosaurus cornalianus*, in which the maxilla is excluded from the internal naris by the posterior process of the premaxilla and the anterolateral process of the palatine (Maisch & Matzke, 1997a). The interpterygoid vacuity is present, but narrow and almost completely occupied by the parasphenoid. One large opening (subtemporal fenestra) in the posterior half of the palate is V-shaped and bounded by the jugal and palatine anteriorly. It anteriorly reaches the middle point of the orbit. The ectopterygoid is absent.

**Vomer**

The paired vomers are complete (Fig. 3). They meet along the midline for most of their extent. The right vomer is more anteriorly dislocated than in natural condition. It is slender with a pointed anterior end, and exceeds the anterior and posterior margins of the internal naris (Fig. 3), forming the entire medial margin of the internal naris. Laterally, the vomer contacts the palatine posteriorly. The anterior half of the vomer is dorsoventrally flat, whereas its posterior half is mediolaterally flat (Figs. 3, 4A and 4B).

**Palatine**

The palatine forms the middle part of the palate. It is a thin plate-like, elongate element with a pointed posterior end. The palatines are separated anteriorly by the vomer and posteriorly by the pterygoid with each other along the mid-line (Fig. 3). The palatine forms the posterior margin of the internal naris. This is different from *Platypterygius* (Kear, 2005), in which the palatine forms the lateral margin of the internal naris, and *Ophthalmosaurus* (Moon & Kirton, 2016), in which the anterior part of the palatine is bifurcated and forms both the lateral and the medial margins of the internal naris. The palatine overlaps the maxilla anterolaterally. It contacts the jugal posterolaterally (Fig. 3), the first observation of this among ichthyosauriforms. The palatine contacts the pterygoid posteromedially along a straight suture. Laterally, it arrives posteriorly aligned to the middle point of the orbit.

Laterally, the anterior half of the palatine is deflected slightly dorsally relative to its posterior half (Fig. 4K). The lateral part of the anterior half of the palatine upturns laterodorsally (Fig. 4L), resulting in its dorsal surface being concave. The anterior half of the palatine has an anterolateral process, as in terrestrial diapsid *Petrolacosaurus* (Reisz, 1981), and ichthyosauriform *Shonisaurus* (Camp, 1980), *Platypterygius* (Kear, 2005), *Ichthyosaurus* (McGowan, 1973) and *Ophthalmosaurus* (Moon & Kirton, 2016). The posterior half of the palatine is nearly flat. It forms the anterior margin of the opening in the posterior half of the palate. Its medial margin is posterolaterally directed and contacts the palatal ramus of the pterygoid.

**Epipterygoid**

Both epipterygoids are completely but dislocatedly preserved (Fig. 3). The anterior margin of the epipterygoid is nearly straight (Figs. 4C and 4D). The dorsal process of the epipterygoid is slender and curved posteriorly. The main body of the epipterygoid is nearly round. Medially, the anterior portion of the main body has a small medial process (Fig. 4D).

**Pterygoid**

The pterygoid is the longest and largest palatal bone. The left pterygoid is preserved in situ, and the right one is dislocated more dorsally (Fig. 3). The anterior extent of the pterygoid is posterior to the external naris. This extent is more posterior than in other ichthyosauriforms whose palates are known, such as in *Mixosaurus* (Maisch & Matzke, 1997a) and *Platypterygius* (Kear, 2005). The posteromedial process of the pterygoid is absent, as in ‘*Mikadocephalus*’ (Maisch & Matzke, 1997b) which may be a subjective junior synonym of *Pessosaurus* (McGowan & Motani, 2003). However, this process is general for ichthyopterygia (Klein et al., 2020). No pterygoidal teeth are detectable, as in previous studies (Zhou et al., 2017; Huang et al., 2019).

The pterygoid is comprised of a palatal ramus and a quadrate ramus. The palatal ramus is dorsoventrally flattened and gradually widens posteriorly (Fig. 3). It contacts the palatine laterally and gets broadest close to the posterior end of the latter. Posterior to the broadest point, the pterygoid has a curved margin laterally. In dorsal view, the palatal ramus has a longitudinal groove across its posterior half with a mediodorsally inturned flange (Figs. 4I and 4J). Posterior to the groove, a small facet marks the articulation with the epipterygoid. The medial margin of the palatal ramus is nearly straight. The transverse flange is developed, and inclined anterolaterally, as previously deduced (Zhou et al., 2017). It is not very prominent in *Cymbospondylus* (Klein et al., 2020). The quadrate ramus is at nearly 90° to the palatal ramus and extends posterolaterally. It contacts the quadrate laterally and has a dorsal process and a posterior process. The dorsal process is plate-like and the posterior process is stout. In medial view, the base of the quadrate ramus has a circular concave fossa for articulation with the basipterygoid process of the basisphenoid (Fig. 4I). A small medial process defines the anteroventral margin of the fossa.

**Quadrate**

Both quadrates are complete (Fig. 3). In lateral view, the quadrate seems to be D-shaped (Fig. 4H). It forms the medial margin of the quadrate foramen (Fig. 4F) which possibly contains a vein from the upper jaw (Romer, 1956), as in *Cymbospondylus* (Fröbisch, Sander & Rieppel, 2010).

The pterygoid process of the quadrate is fan-shaped and contacts the quadrate process of the pterygoid medially. The dorsal half of its articular surface for the quadrate process of the pterygoid is flat, whereas the ventral half is concave (Fig. 4G), as in ‘*Mikadocephalus*’ (Maisch & Matzke, 1997b). The medial surface of the pterygoid process is different from that of *Shonisaurus* which bears a circular pit for the proximal tip of the stapes (Camp, 1980). In lateral view, the ventral half of the anterior margin of the pterygoid process is nearly vertical, and possibly for contact with the epipterygoid. The anteroventral margin of the pterygoid process is nearly straight and forms an obtuse angle with its anterior margin (Figs. 4G and 4H). The quadrate head articulates with the squamosal dorsolaterally and the supratemporal dorsomedially (Figs. 4G and 4H). In dorsal view, it is smooth and curved posteromedially (Fig. 4E). Ventral to the quadrate head, there is a ridge for articulation with the squamosal at the posterolateral margin of the quadrate (Fig. 4H). Anterior to the ridge, a depression on the quadrate marks the point of the attachment for *M. adductor mandibulae posterior*. Posterior to the ridge, the quadrate is exposed laterally. The articular condyle of the quadrate is stout and extends laterally. Dorsally, it has a concave surface for articulating with the quadratojugal (Fig. 4E). Ventrally, it has a medioventrally directed surface for articulating with the articular.

**Palatal dentition**

**Figure 5:**
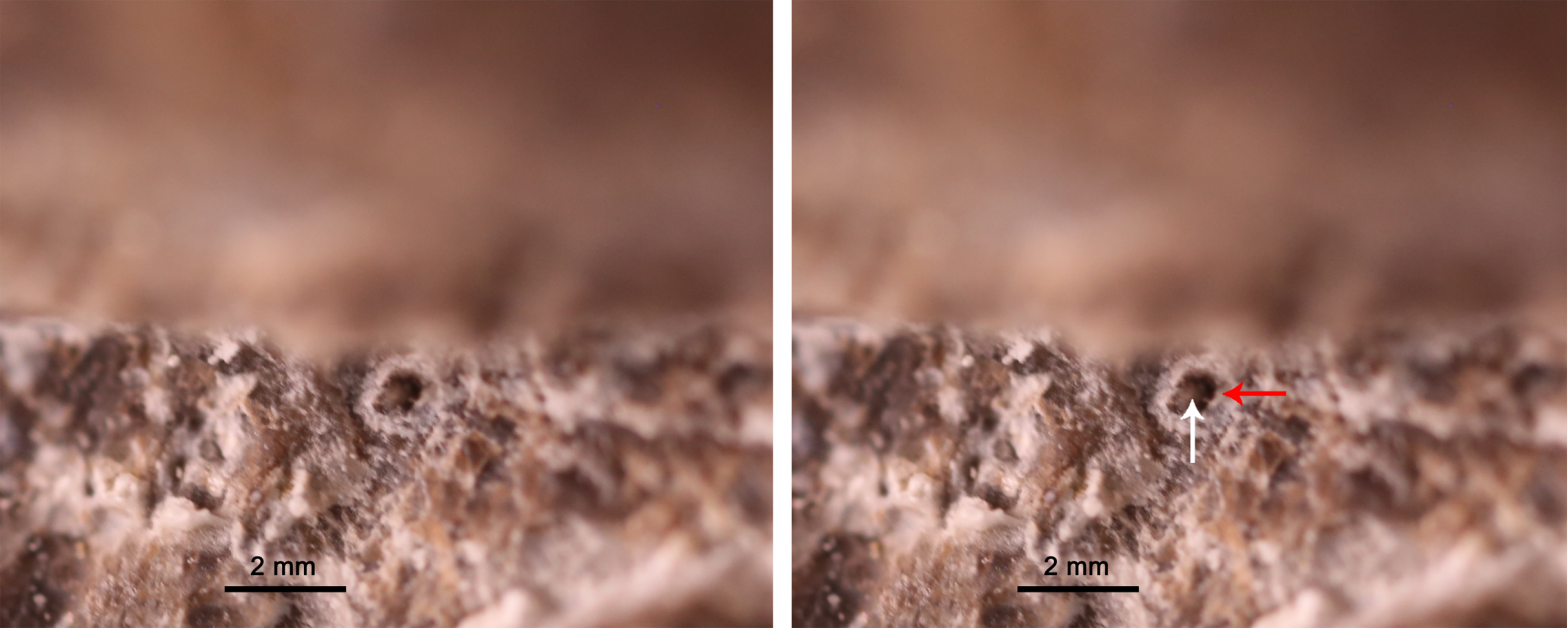
Exposed left third palatal denticle of GMPKU-P-3086. White and read arrows point to the denticle and denticle depression respectively.

Seven tooth-like projections are closely attached to each palatine (Fig. 3). However, it cannot be confirmed that they are firmly implanted in the palatine (Video S3; https://www.morphosource.org/concern/media/000355471?locale=en). These projections seem to be bilaterally symmetrical and organized in a single row on both sides (Fig. 3). The height and width of one projection are both approximately 1 mm. The length of the row of the projections is 16 mm (Table 1). The first projection is located just at the level of the posterior end of the maxilla. The left third projection can be observed directly, which seems to be attached to a circular depression (Fig. 5). The bases of the projections lie ventral to those of the maxillary teeth because the posterior half of the palatine extends ventrally below the level of the maxilla. The weak enamel and dentine on an individual tooth-like projection can be observed through the horizontal sectional video of the right row (Video S3; https://www.morphosource.org/concern/media/000355471?locale=en), but no clear pulp cavity can be observed under current scanning spatial resolution (34.9 μm).

**Table 1:** Measurements (in mm) of the palate of *Chaohusaurus brevifemoralis* (GMPKU-P-3086).

	Left side	Right side
Length of the choana	14.8	14.4
Length of the vomer	36	36
Length of the palatine	42	42
Height of the epipterygoid	17	16
Height of the third palatal denticle	0.9	1
Width of the third palatal denticle	0.8	0.8
Length of the palatal denticle row	16	16
Distance between the pterygoid anterior to the quadrate ramus of the pterygoid and the parietal	~29	~30

## Discussion

Specimen GMPKU-P-3086 reveals a complete palate of Early Triassic ichthyosauriforms for the first time based on CT scanning and 3D reconstruction. It provides new morphological characters of *Chaohusaurus brevifemoralis* that may shed light on palatal evolution and phylogeny of basal ichthyosauriforms. We discuss these features regarding the suborbital fenestra, palatal denticles, and other structures as below.

### A the suborbital fenestra

**Figure 6:**
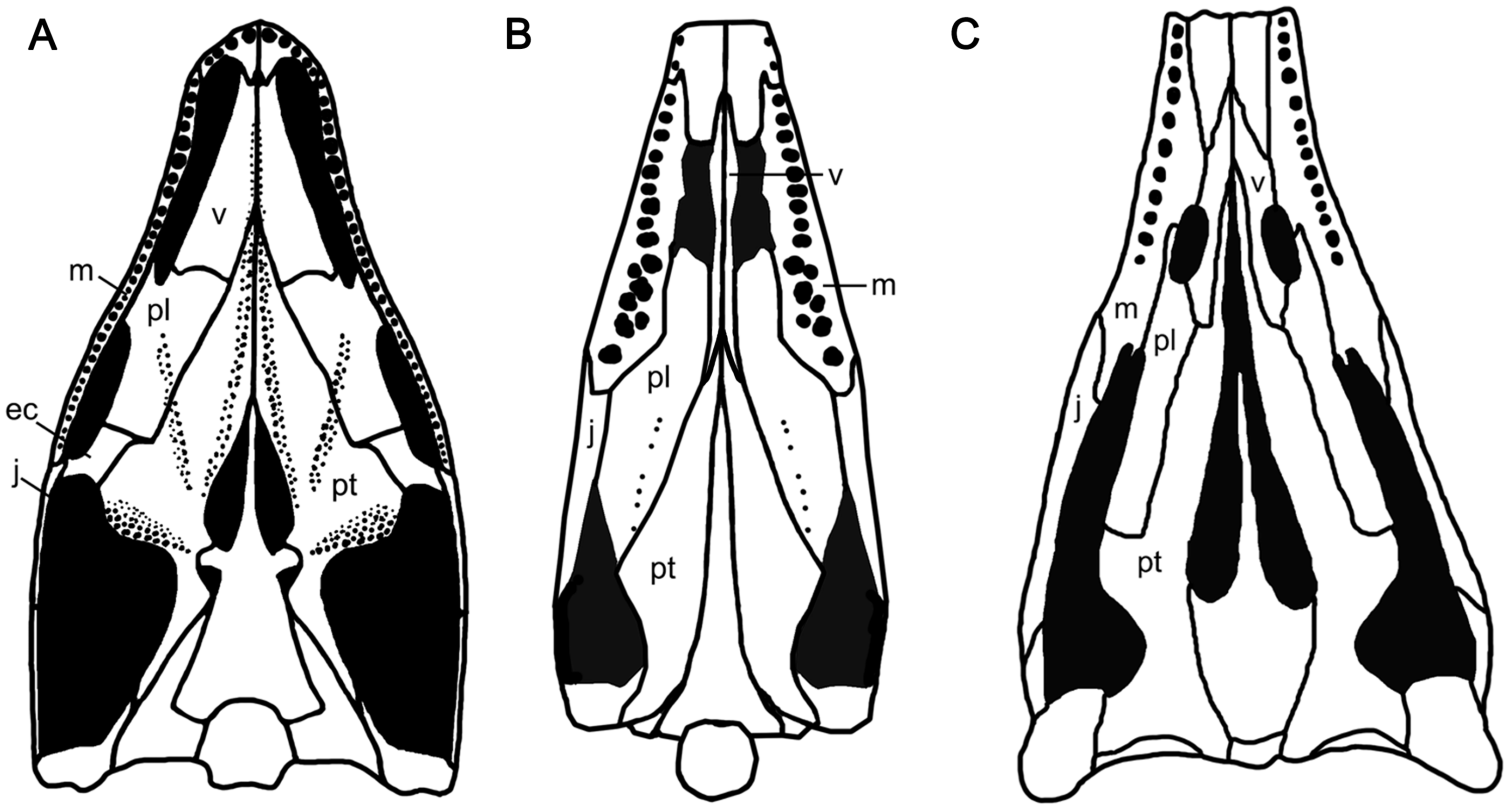
Palatal morphology of select basal diapsid and ichthyosauriforms. (A) *Petrolacosaurus* (after Reisz, 1981). (B) *Chaohusaurus brevifemoralis*. (C) *Ichthyosaurus* (after Romer, 1956). Abbreviations: ec, ectopterygoid; j, jugal; m, maxilla; pl, palatine; pt, pterygoid; v, vomer.

The diapsid palate generally has suborbital and subtemporal fenestrae, separated by the ectopterygoid, such as in basal diapsid *Petrolacosaurus* (Reisz, 1981; Fig. 6A). The presence of the suborbital fenestra, which is associated with the *Musculus pterygoideu*s (Tarsitano et al., 2001), is a synapomorphy in diapsids (Benton, 1985). Typically, it is bounded by the maxilla, palatine and ectopterygoid (Gaffney, 1980; Callaway, 1989; Fig. 6A), and can be observed dorsally through the orbit, such as in basal diapsid *Petrolacosaurus* (Reisz, 1981), *Youngina* and *Claudiosaurus* (Carroll, 1981). Callaway (1989) and Massare & Callaway (1990) suggested that the opening interpreted as the subtemporal fenestra in the ichthyosaurian palate, bordered anteriorly by the maxilla and palatine, was a merged suborbital and subtemporal fenestrae resulting from the reduction and loss of the ectopterygoid (Fig. 6C). This has been confirmed in *Phalarodon atavus* (Maisch & Matzke, 1998a, 2001), *Callawayia wolonggangense* (Chen, Cheng & Sander, 2007), *Ophthalmosaurus* (Moon & Kirton, 2016), *Platypterygius* (Kear, 2005) and *Ichthyosaurus* (McGowan, 1973; McGowan & Motani, 2003; Fig. 6C). In this paper, we refer to the opening in the palate of ichthyosaurians as modified subtemporal fenestra to differentiate it from that of basal diapsids.

The anatomy of the modified subtemporal fenestra has never been reported in Early Triassic ichthyosauriforms because the specimens showing their palatal anatomy are extremely limited as mentioned above. Huang et al. (2019) deduced that the suborbital fenestra was absent in *Chaohusaurus brevifemoralis* based on the ventrally continuously exposed palatine and pterygoid of GMPKU-P-3086, which are laterally concealed by the mandibles. Here using the CT scanning, one large opening is clearly present in the posterior half of the palate of GMPKU-P-3086, which is bordered anteriorly by the palatine and jugal (Fig. 6B). Unlike the modified subtemporal fenestra in Ichthyosauria, the maxilla does not participate in forming the anterior margin of the opening. This is caused by the lateral and posterior enlargement of the palatine that contacts the jugal directly, excluding the maxilla from forming the anterior margin of this opening (Fig. 6B). Dorsally, the anterior part of the opening can be observed through the posterior half of the orbit (Fig. 2).

The different anatomy of the opening in the palate of *C. brevifemoralis* could be interpreted in two ways based on the two possible states of the suborbital fenestra. First, it could be homologous to the modified subtemporal fenestra of Ichthyosauria. This opening is formed by merging the subtemporal fenestra and the suborbital fenestra due to the loss of the ectopterygoid. The difference between the two anatomies between *C. brevifemoralis* and Ichthyosauria is whether the lateral and posterior enlargement of the palatine excludes the maxilla from bordering the opening. Second, the unique anatomy of the palatal opening of *C. brevifemoralis* is derived from the subtemporal fenestra of basal diapsids by closing the suborbital fenestra. The suborbital fenestra of *C. brevifemoralis* is closed due to the enlargement of the palatine and the loss of the ectopterygoid. Therefore, the palatine contacts the jugal directly, which is first observed in *C. brevifemoralis*. However, the second interpretation raises a new question on whether the suborbital fenestra was closed among all basal ichthyosauriforms or only within *C. brevifemoralis*. Currently, it is difficult to verify due to the lack of information on the Early Triassic ichthyosauriform palates. If additional Early Triassic materials are found in the future, whose suborbital fenestra is closed due to the enlargement of the palatine with a definitive ectopterygoid, the second interpretation will be supported. It means that the ‘suborbital fenestra’ of derived ichthyosauriforms is possibly secondarily evolved by the reduction of the palatine which makes room for the maxilla to participate in the formation of the anterior margin of the modified subtemporal fenestra (Fig. 6C).

Additionally, the closure of the suborbital fenestra convergently appeared in other diapsids in different ways. In the rhynchocephalian *Priosphenodon*, it is caused by the expansion of the ectopterygoid (Apesteguía & Novas, 2003). In the basal sauropterygian *Panzhousaurus* (Lin et al., 2021) and *Dianmeisaurus* (Shang & Li, 2015), the expanded palatine results in the closure of this fenestra with a reduced ectopterygoid. In the plesiosaurian *Cryptoclidus* (Brown & Cruickshank, 1994) and *Ophthalmothule* (Roberts et al., 2020), the expanded palatine also results in the closure of this fenestra but without definitive information of the ectopterygoid. Before more information is obtained on the palatal morphology among basal ichthyosauriforms, it is temporally difficult to further interpret the anatomy of the palatal opening in *Chaohusaurus brevifemoralis*.

### B palatal denticles

So far, palatal teeth have been reported in *Utatsusaurus* (Motani, 1997; Motani, 1999; Cuthbertson, Russell & Anderson, 2013b) and ‘*Wimanius*’ (Maisch & Matzke, 1998b) which is of doubtful validity (McGowan & Motani, 2003) among ichthyosauriforms. Here, the palatal teeth of the two taxa are referred to as denticles because they are small, about 1 mm in height. However, the denticles in both genera are controversial because their palates are fragmentary (Motani, 1997; McGowan & Motani, 2003). In *Utatsusaurus*, two small denticles are present on the transverse flange of the pterygoid of the referred specimen (UHR 30691). Motani (1997) suggested that they were displaced germ teeth of marginal dentition or vestigially pterygoidal dentition. Shortly thereafter, Motani (1999) indicated that they were vestigial denticles on the pterygoid but without giving any explanation. Cuthbertson, Russell & Anderson (2013b) found that one of the two denticles was already broken and no longer in situ while the other was undamaged and in situ, and argued that the pterygoid denticles of *Utatsusaurus* were indeed present. After personal communication with R. Motani, Matsumoto & Evans (2017) suggested that re-examination is still needed to confirm the presence of the pterygoid denticles in *Utatsusaurus*. The palatal denticles attached to the palatine or pterygoid of ‘*Wimanius*’ are also under debate (Maisch & Matzke, 1998b). A single row of denticles on the palatine was reported in ‘*Wimanius*’ by Maisch & Matzke (1998b), but later studies questioned the identification of the palatine and suggested that this bone bearing the denticles is possibly a broken pterygoid (Motani, 1999; McGowan & Motani, 2003). The controversy exists because of the poorly preserved palatine and pterygoid in the holotype of ‘*Wimanius*’.

With the aid of CT scanning, two rows of tooth-like projections seem to be observed on the paired palatines of *Chaohusaurus brevifemoralis* (GMPKU-P-3086). They can be ruled out as small minerals because they appear bilaterally symmetrical and organized in a single row on both sides. Therefore, they are most likely biological structures. Whether these projections are firmly implanted in the palatine cannot be confirmed although they appear closely attached to this bone. These projections could be either marginal germ teeth or palatal teeth. With the current scans, it is unlikely they are displaced germ teeth of marginal dentition because their enamel has lower density than the enamel of the marginal teeth (Video S2; https://www.morphosource.org/concern/media/000355466?locale=en; Video S3; https://www.morphosource.org/concern/media/000355471?locale=en), considering that the marginal germ teeth also have the same dense enamel as the marginal teeth in reptiles (Romer, 1956). Here these tooth-like projections are conservatively referred to as denticles rather than teeth because no pulp cavity can be observed under current scanning resolution and they are small, about 1 mm in height.

The palatal denticles can be present on the vomer, pterygoid, palatine and parasphenoid in basal diapsids (Matsumoto & Evans, 2017). Based on the position of the denticles on the reconstruction image, we can rule out the possibility that they belong to the vomer and parasphenoid, and the bone bearing the denticles could be either pterygoid or palatine. In terms of preservation, they appear to be bilaterally symmetrical, and are attached to the palatine, preserved in situ and organized as a single row on each palatine in GMPKU-P3086. Therefore, they most likely belong to the palatine in *C. brevifemoralis*. However, the lack of critical evidence that these denticles are firmly implanted in the palatine cannot totally preclude the possibility that they belong to the pterygoid. Above all, we think that a single row of denticles is present on each side of the palate in *C. brevifemoralis*.

The pterygoid denticles of *Utatsusaurus*, the palatal denticles of *C. brevifemoralis* and ‘*Wimanius*’ indicate that palatal denticles are plesiomorphically present in basal ichthyosauriforms, which are absent in other ichthyosauriforms. Due to preservational reasons, the complete ventral information of the palate remains rarely known among basal ichthyosauriforms. Re-evaluation of the palatal denticles in other basal ichthyosauriforms is needed based on well-preserved specimens and the application of techniques such as CT scanning. Also, more materials are needed to confirm the anatomy of the bone bearing denticles in *C. brevifemoralis* and ‘*Wimanius*’.

### C other notable palatal characters

The position of the vomer relative to the internal naris in ichthyosauriforms is different from that of basal diapsids, in which the vomer does not exceed the anterior and posterior margins of the internal naris, such as in *Youngina* (Carroll, 1981) and *Petrolacosaurus* (Reisz, 1981; Fig. 6A). In ichthyosauriforms, the vomer exceeds the anterior and posterior margins of the internal naris, such as in *Chaohusaurus brevifemoralis* (Fig. 6B), and the ichthyopterygian *Cymbospondylus petrinus* (Merriam, 1908), *Mixosaurus cornalianus* (Maisch & Matzke, 1997a), *Phalarodon atavus* (Maisch & Matzke, 2001), *Callawayia wolonggangense* (Chen, Cheng & Sander, 2007), *Ophthalmosaurus icenicus* (Moon & Kirton, 2016), *Platypterygius longmani* (Kear, 2005) and *Ichthyosaurus* spp. (McGowan, 1973; Fig. 6C). However, the vomer morphology remains unknown in Nasorostra and Hupehsuchia. Therefore, the vomer exceeding the anterior and posterior margins of the internal naris represents a synapomorphy of a taxon, which needs to be confirmed in the future, including the *Chaohusaurus* and Ichthyopterygia.

The position of the anterior end of the pterygoid relative to the internal naris has a trend of anterior displacement through the evolution of ichthyosauriforms. Anteriorly, the pterygoid is located posterior to the internal naris in *Chaohusaurus brevifemoralis* (Fig. 6B), and *Cymbospondylus* spp. (Merriam, 1908; Fröbisch, Sander & Rieppel, 2010; Klein et al., 2020). In mixosaurs, the pterygoid reaches anteriorly up to the posterior margin of the internal naris in *Mixosaurus cornalianus* (Maisch & Matzke, 1997a) and the middle part of the internal naris in *Phalarodon atavus* (Maisch & Matzke, 1998a, 2001). In parvipelvians, the pterygoid extends anteriorly to the anterior margin of the internal naris in *Platypterygius* (Kear, 2005) and exceeds the anterior margin of the internal naris in *Ichthyosaurus* (McGowan, 1973; Fig. 6C) and *Ophthalmosaurus* (Moon & Kirton, 2016).

The epipterygoid is scarcely known among ichthyosauriforms. Previously, the ossified epipterygoid has only been reported in *Ichthyosaurus* (McGowan, 1973) and ‘*Mikadocephalus*’ (Maisch & Matzke, 1997b). The shape of the epipterygoid of *Chaohusaurus brevifemoralis* is different from that of *Ichthyosaurus* (McGowan, 1973). The anterior and posterior margins of the epipterygoid in *C. brevifemoralis* are straight and curved respectively, while they appear in the opposite way in *Ichthyosaurus* (McGowan, 1973). Particularly, the length of the epipterygoid of *C. brevifemoralis* is shorter than the distance between the pterygoid anterior to the quadrate ramus and the parietal (Table 1). No articular facet is observed on the parietal for the epipterygoid in *C. brevifemoralis*, while it is present in *Ichthyosaurus* (McGowan, 1973). Therefore, the epipterygoid of *C. brevifemoralis* possibly contacts the parietal by a soft tissue. In *Ophthalmosaurus* (Moon & Kirton, 2016) and *Platypterygius* (Kear, 2005), the epipterygoid fails to ossify, but the pterygoid and parietal have the articular facets for the epipterygoid. The different morphology of the epipterygoid within these four genera confirms that the degree of ossification in the epipterygoid varies among ichthyosauriforms, as previously suggested (Moon & Kirton, 2016).

The ectopterygoid is absent in Ichthyopterygia but present in basal diapsids and hupehsuchian *Hupehsuchus* (Carroll & Dong, 1991). The absence of the ectopterygoid was considered as a synapomorphy of Ichthyopterygia based on former phylogenetic analyses (Motani, 1999; Ji et al., 2015). Particularly, a possible ectopterygoid has been reported in *Cymbospondylus duelferi* (Klein et al., 2020) among ichthyopterygia. In *Chaohusaurus*, the ectopterygoid has been considered as absent and the absence of the ectopterygoid has been only known in *C. chaoxianensis* to date (Ji et al., 2015; Huang et al., 2019). The absence of the ectopterygoid, however, has never been confirmed on published specimens of *C. chaoxianensis* because this part of the skull was hardly exposed. Here, the specimen (GMPKU-P-3086) clearly shows the absence of the ectopterygoid in *C. brevifemoralis* under the aid of CT scanning. This finding confirms the absence of the ectopterygoid in *Chaohusaurus* for the first time. Following a recent phylogenetic relationship of Ichthyosauriformes that *Chaohusaurus* is the sister group of Ichthyopterygia (Huang et al., 2019), this finding definitely indicates that the absence of the ectopterygoid is diagnostic of a more inclusive clade including the *Chaohusauru*s and Ichthyopterygia. So far the status of the ectopterygoid has not been verified in Nasorostra.

## Conclusion

Under the assistance of CT scanning, specimen GMPKU-P-3086 reveals new palatal features of *Chaohusaurus brevifemoralis*. The palatine contacts the jugal directly, which is first observed among ichthyosauriforms. A single row of denticles is present on each side of the palate. The vomer exceeds the anterior and posterior margins of the internal naris. The pterygoid is posterior to the internal naris. The epipterygoid is present and the ectopterygoid is absent.

## Additional Information and Declarations

### Data Availability

The following information was supplied regarding data availability: The media is available at MorphoSource: Skull (Palate), 000355461, DOI 10.17602/M2/M355461; Skull (Palatal CT Data), 000355466, DOI 10.17602/M2/M355466; Skull (Right Palatal Denticles), 000355471, DOI 10.17602/M2/M355471.
